# Paeonol Attenuates Atherosclerosis by Inhibiting Vascular Smooth Muscle Cells Senescence via SIRT1/P53/TRF2 Signaling Pathway

**DOI:** 10.3390/molecules29010261

**Published:** 2024-01-04

**Authors:** Min Zhou, Xiaolin Ma, Menglong Gao, Hongfei Wu, Yarong Liu, Xiaoyan Shi, Min Dai

**Affiliations:** 1 College of Pharmacy, Anhui University of Chinese Medicine, Hefei 230012, China; zhoumin10161115@163.com (M.Z.); mxl19981226@163.com (X.M.); gmledu@163.com (M.G.); wuhongfei@ahtcm.edu.cn (H.W.); lyr1209@ahtcm.edu.cn (Y.L.); 2 Anhui Key Laboratory for Research and Development of Traditional Chinese Medicine, Hefei 230012, China

**Keywords:** paeonol, atherosclerosis, vascular smooth muscle cells, cell senescence, SIRT1

## Abstract

Atherosclerosis is a chronic inflammatory disease leading to various vascular diseases. Vascular smooth muscle cell (VSMC) senescence promotes atherosclerotic inflammation and the formation of plaque necrosis core, in part through telomere damage mediated by a high-fat diet. Our previous research found that paeonol, a potential anti-inflammatory agent extracted from Cortex Moutan, could significantly improve VSMCs dysfunction. However, the impact of paeonol on the senescence of VSMCs remains unexplored. This study presents the protective effects of paeonol on VSMCs senescence, and its potential activity in inhibiting the progression of atherosclerosis in vivo and in vitro. Sirtuin 1 (SIRT1) is a nuclear deacetylase involved in cell proliferation, senescence, telomere damage, and inflammation. Here, SIRT1 was identified as a potential target of paeonol having anti-senescence and anti-atherosclerosis activity. Mechanistic studies revealed that paeonol binds directly to SIRT1 and then activates the SIRT1/P53/TRF2 pathway to inhibit VSMCs senescence. Our results suggested that SIRT1-mediated VSMCs senescence is a promising druggable target for atherosclerosis, and that pharmacological modulation of the SIRT1/P53/TRF2 signaling pathway by paeonol is of potential benefit for patients with atherosclerosis.

## 1. Introduction

Atherosclerosis is the common pathological mechanism underlying many cardiovascular and cerebrovascular diseases with limited therapeutic options [1]. The formation of plaques and necrotic cores is promoted by vascular smooth muscle cells (VSMCs), which derive from the medial layer of the blood vessel and play a central role in atherosclerosis [2]. In particular, VSMC senescence and severe inflammation are observed in both the atherosclerotic patient and in mice models, resulting in the progression of atherosclerosis [3]. Recently, much attention has focused on telomere damage-based senescence of VSMCs in atherosclerosis [4,5]. However, the potential targets and mechanisms of VSMC senescence remain unknown.

Sirtuin 1 (SIRT1) is a member of the Sirtuin family of class III histone deacetylase enzymes, which plays a critical role in modulating cell senescence [6], cell proliferation [7], and inflammation responses [8]. Extensive studies have shown that SIRT1 overexpression protects human vessels against stress senescence of VSMCs stimulated by inflammation [9,10], thus improving the normal function of VSMCs, inhibiting vascular inflammation and plaque formation [3]. At the mechanistic level, p53 is a known senescence-promoting protein and is a downstream transcription factor regulated by SIRT1 [11]. Concomitantly, P53, as a transcription factor inhibiting telomere repeat binding factor-2 (TRF2) expression, is associated with telomere shortening, and induces cell senescence and inflammation [12]. Importantly, VSMC-specific SIRT1 or TRF2 mutant mice show increased atherosclerosis progression, which is associated with increased cell senescence [12,13]. Therefore, SIRT1 may be an attractive therapeutic target for atherosclerosis. There is an urgent need to identify small-molecule drugs that selectively target SIRT1 for the treatment of atherosclerosis.

Paeonol, a bioactive compound extracted from the bark of *Paeonia suffruticosa* Andr. has a long history of clinical application as a potential anti-inflammatory agent in inflammation-related indication [14]. It is noteworthy that paeonol has been approved by the China Food and Drug Administration for the treatment of inflammatory diseases, and has a significant effect of relieving inflammatory response [14,15]. Our previous studies have demonstrated that paeonol has potential inhibitory effects in atherosclerosis progression and VSMC apoptosis based on its anti-inflammatory activity [16,17]. Furthermore, paeonol has been reported to significantly delay vascular endothelial cell senescence, and SIRT1 could be an important target for paeonol [18]. However, the effect and direct targets of paeonol on the senescence of VSMCs in the process of atherosclerosis has not yet been evaluated.

In the present study, we explored the effects of paeonol on atherosclerosis progression in vivo and on tert-butyl hydroperoxide (t-BHP)-induced senescence of VSMCs in vitro, with a specific focus on the mechanisms by which paeonol attenuates telomere damage in VSMCs. Our results revealed that paeonol is a novel SIRT1-regulated molecule that attenuates telomere damage and inhibits VSMCs senescence through the SIRT1/P53/TRF2 signaling pathway.

## 2. Results

### 2.1. Paeonol Attenuates the Development of HFD-Induced Atherosclerosis in ApoE^−/−^ Mice

**Figure 1:**
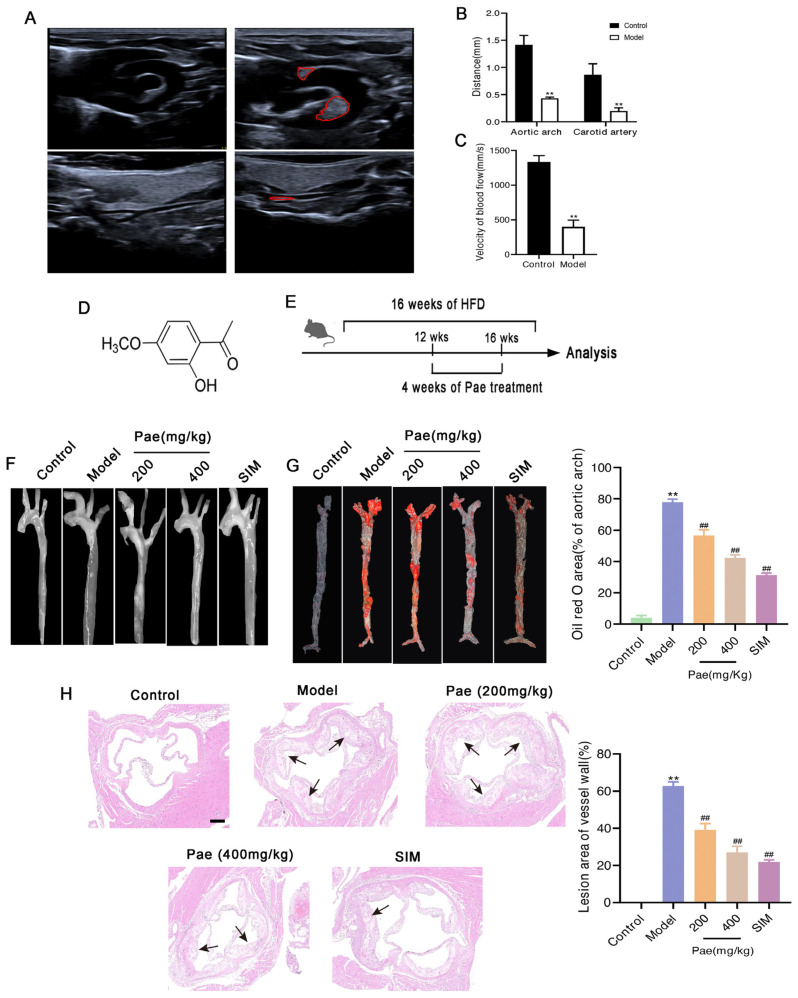
Paeonol administration mitigates the progression of atherosclerosis in ApoE^−/−^ mice. (**A**) Ultrasound images of the aortic arch and abdominal aorta in each group (the area circled by the red line indicates plaque formation). (**B**) Distance and (**C**) velocity of blood flow of mice in each group. (**D**) The chemical structure of paeonol. (**E**) Experimental design of C57BL/6J, ApoE^−/−^, and ApoE^−/−^ + paeonol (400 and 200 mg/kg/day by intragastric administration once daily for 4 weeks). (**F**) Photographs of the aortic arches in each group. (**G**) En face Oil-Red O staining of the aortas in each group. Red: Lipid plaque deposition in intima. (**H**) H&E staining of the aortic sinus of mice in each group. Scale bar, 100 μm. The results are expressed as mean ± SD, ** *p* < 0.01 versus control; ^##^ *p* < 0.01 versus the model.

To verify whether the atherosclerotic mice model was established successfully, plaque formation and the velocity of blood flow in the aorta of HFD-induced mice for 12 weeks were monitored by ultrasound imaging. As shown in Figure 1A, ultrasound imaging results confirmed that the model group showed significant plaque areas in the aortic arch and abdominal aorta compared to the control group. Similarly, in the model group, the distance from the lumen of the aorta was reduced and the blood flow velocity was significantly slowed, suggesting that the intima of the blood vessels of the mice was thickened and blood flow was blocked (Figure 1B,C).

To determine the effects of paeonol protection against atherosclerosis in mice, ApoE^−/−^ mice were fed HFD weeks to establish the atherosclerosis model. After 12 weeks, mice were supplemented with paeonol (400, and 200 mg/kg/day by intragastric administration once daily for 4 weeks) according to our previous studies (Figure 1D,E). Obviously, as shown in Figure 1F, photographs of the aortic arches suggested that paeonol treatment significantly protected ApoE^−/−^ mice from atherosclerosis. Furthermore, ORO staining showed that Pae markedly reduced lipid deposition in the aortic intima (Figure 1G). H&E staining (Figure 1H) revealed a smooth aortic intima in C57BL/6J mice, a thin tunica intima and no plaque formation. Meanwhile, typical plaques of atherosclerosis plaques were observed in the aorta of the model group. After treatment with paeonol or simvastatin, the plaque area of atherosclerosis in the aortic intima decreased compared to the model group. These findings demonstrated that paeonol treatment alleviated the development of atherosclerosis in ApoE^−/−^ mice.

### 2.2. Paeonol Prevents Vascular Senescence in ApoE^−/−^ Mice

**Figure 2:**
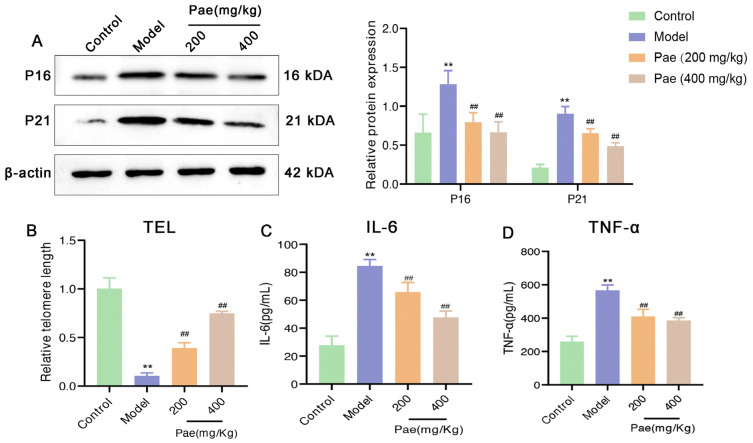
Paeonol reduces atherosclerosis-induced vascular senescence. (**A**) Paeonol significantly down-regulated the protein expression of P16 and P21 by Western blotting analysis. (**B**) The mRNA expression of TEL was increased after paeonol treatment by qRT-PCR. (**C**,**D**) Typical levels of SASP (TNF-α and IL-6) levels in serum and aortas of mice were decreased after paeonol treatment. The results are expressed as mean ± SD, ** *p* < 0.01 versus control; ^##^ *p* < 0.01 versus model.

Evidence suggested that senescent cells and a more severe inflammatory response were found in the aorta of atherosclerosis mice and could further accelerate the pathological progression of atherosclerosis [3,19]. Accumulating evidence showed that senescent cells that express markers such as SA-β-gal, p16, and p21 contribute to inflammation by secreting the senescence-associated secretory phenotype (SASP) [6]. To determine whether paeonol protects against senescence in tissues of the aorta, we further detected the expression of senescence-associated proteins in aorta by Western blotting. In paeonol-treated mice, we found a strongly decreased expression of aortic P16 and P21 compared to the model group (Figure 2A). Furthermore, paeonol decreased the expression of TEL mRNA in the aorta of atherosclerosis mice (Figure 2B). Senescent cells produce higher levels of typical SASP compared to normal cells, which can further promote local vascular inflammation and plaque progression. According to our results, typical levels of SASP (TNF-α, and IL-6) in serum from atherosclerosis mice were higher, while paeonol significantly decreased TNF-α and IL-6 compared with the model group (Figure 2C,D). Together, these data suggested that paeonol alleviated the vascular senescence and the inflammatory reaction process that occurs during atherosclerosis.

### 2.3. Paeonol Attenuates VSMCs Senescence and Regulates the SIRT1 Pathway in ApoE^−/−^ Mice

**Figure 3:**
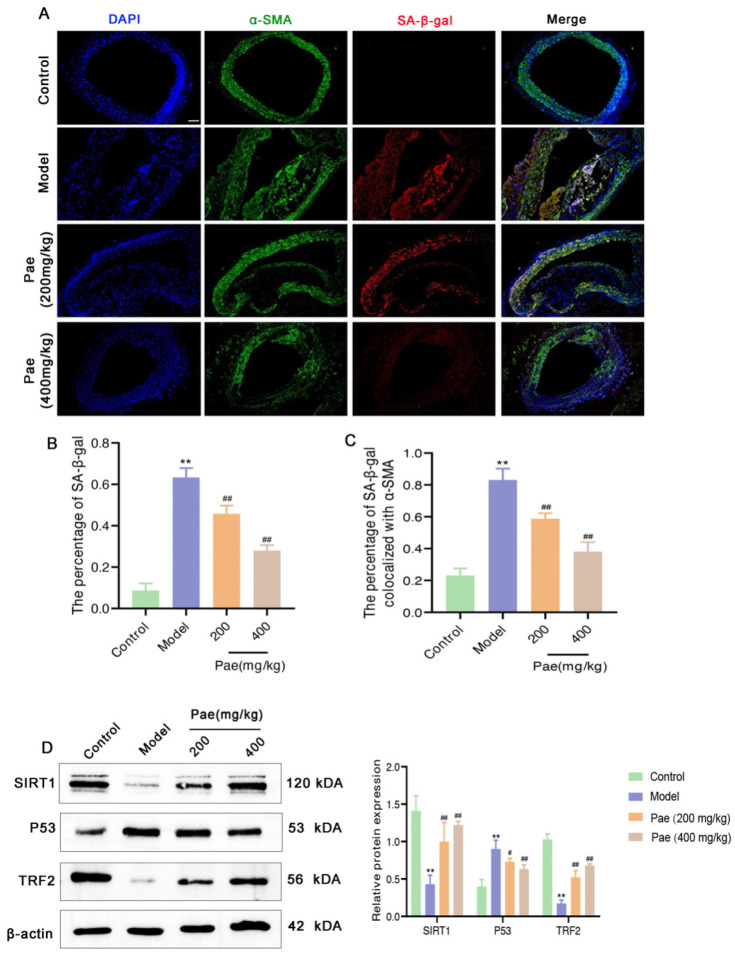
Paeonol inhibits the senescence of VSMCs and activates SIRT1 signaling in ApoE^−/−^ mice. (**A**) Representative α-SMA and SA-β-gal immunofluorescence in artery intracuff lesions after treatment with paeonol in ApoE^−/−^ mice. DAPI was used for nuclear staining. Scale bar, 100 μm. Paeonol decreased the percentage of (**B**) SA-β-gal and (**C**) SA-β-gal colocalized with α-SMA in artery intracuff lesions of ApoE^−/−^ mice. (**D**) SIRT1, P53, and TRF2 protein levels were down-regulated after paeonol treatment by Western blotting analysis. The results are expressed as mean ± SD, ** *p* < 0.01 versus control; ^#^ *p* < 0.05, ^##^ *p* < 0.01 versus model.

Our data showed that paeonol could inhibit vascular senescence and inflammation in the mouse aorta. To explore the effects of paeonol on VSMC senescence in the aorta of ApoE^−/−^ mice, we observed the fluorescent intensity of α-SMA and SA-β-gal by immunofluorescence staining. As shown in Figure 3A,B, the fluorescent intensity of SA-β-gal was dramatically decreased in the aortas of paeonol-treated mice compared with the model group. More importantly, by calculating the fluorescence colocalization coefficient (Figure 3A,C), we found that the expression of SA-β-gal in VSMCs in the aorta of atherosclerotic mice increased significantly, suggesting the presence of senescent VSMCs in atherosclerotic plaques. Paeonol intervention significantly inhibited VSMC senescence in plaques. This might be one important reason why paeonol can inhibit the development of atherosclerosis in ApoE^−/−^ mice.

SIRT1 signaling activation has been shown to inhibit cell senescence [9]. Consistent with the expected results, paeonol intervention significantly increased protein expression levels of SIRT1 and TRF2, whereas it decreased P53 expression, suggesting that paeonol activated the SIRT1 signaling pathway in the aorta of atherosclerotic mice (Figure 3D). To clarify the inhibitory effect and potential mechanism of paeonol on VSMC senescence, we extracted VSMCs from mice for primary culture and further in vitro study.

### 2.4. Paeonol Delays the Senescence of t-BHP-Injured VSMCs

**Figure 4:**
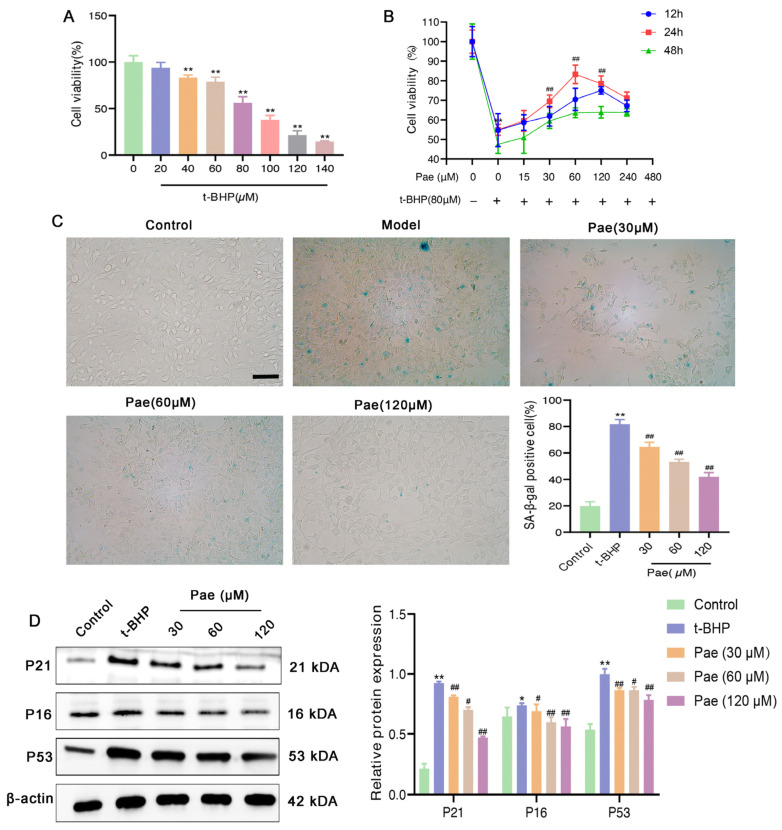
Paeonol delays t-BHP stimulated senescence of VSMCs. Effects of t-BHP (**A**) and paeonol (**B**) on the activity of VSMCs by the CCK-8 assay. (**C**) After treatment with paeonol, cellular senescence was significantly reduced by SA-β-gal staining (senescent cells were stained blue). Scale bar, 10 μm. (**D**) The expression level of senescence-associated proteins (P21, P53, and P16) in t-BHP stimulated VSMCs was significantly down-regulated by paeonol. The results are expressed as mean ± SD, * *p* < 0.05, ** *p* < 0.01 versus control; ^#^ *p* < 0.05, ^##^ *p* < 0.01 versus t-BHP.

VSMC senescence has been reported to be closely related to HFD-induced atherosclerosis and vascular inflammation [20]. To investigate the role of paeonol in VSMC senescence in vitro, VSMCs were isolated from mouse C57BL/6J arteries as described in our previous study [16]. As a classic stimulator of cell senescence, t-BHP can induce stress-induced senescence in a variety of cells [21,22]. In the present study, VSMCs were stimulated with 80 μM t-BHP for 24 h to establish VSMC senescence models (Figure 4A), and then treated with a range of different concentrations of paeonol (15, 30, 60, 120, 240 μM) for 24, 48, and 72 h, respectively. We found that increasing concentrations of paeonol (15–240 μM) gradually eliminated the inhibitory effects of t-BHP induced on the viability of VSMCs (Figure 4B). VSMCs were treated with t-BHP with or without a low (30 μM), a medium (60 μM), or a high dose (120 μM) of paeonol for 24 h for subsequent experiments. Next, we used SA-β-gal staining to detect the degree of cell senescence (Figure 4C) and found that VSMC senescence (cells in blue) was increased in the t-BHP stimulated group, while paeonol treatment attenuated the increase in SA-β-gal in VSMCs induced by t-BHP. Expressions of P21, P53, and P16 in paeonol-treated VSMCs were also significantly lower than in the model group (Figure 4D). Together, these results suggested that paeonol treatment could significantly inhibit VSMC senescence.

### 2.5. Paeonol Inhibits SIRT1-Mediated Telomere Damage and Partially Suppresses Inflammation in Senescent VSMCs

**Figure 5:**
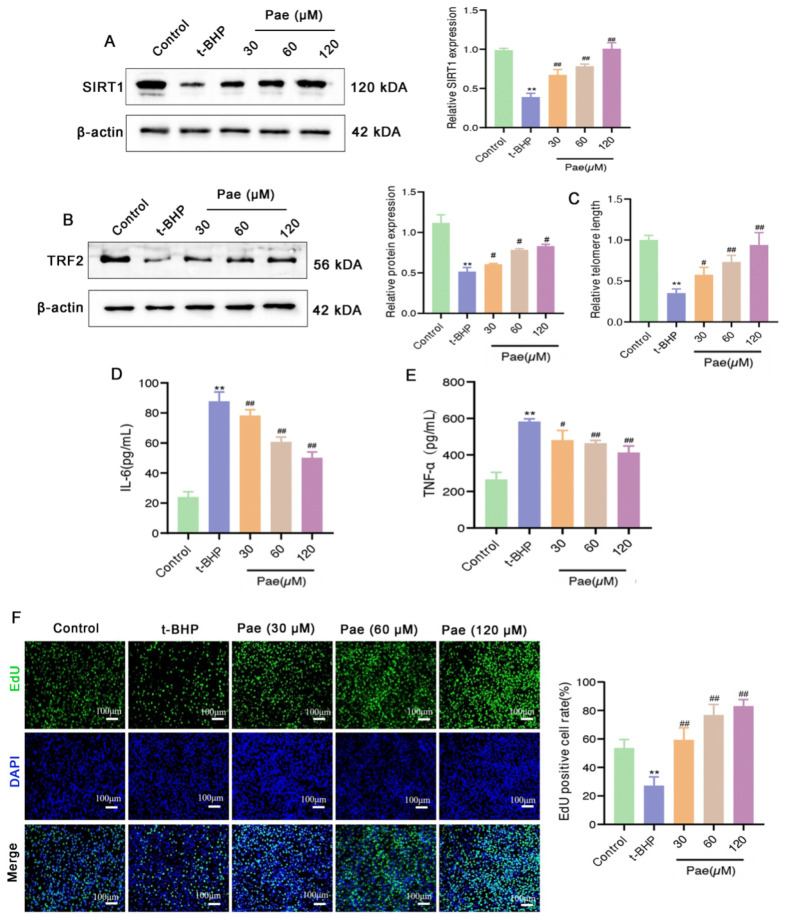
Paeonol increases the expression of SIRT1 and attenuates telomere damage in t-BHP-induced senescent VSMCs. VSMCs were exposed to t-BHP and then treated with paeonol, protein expression levels of SIRT1 (**A**) and TRF2 (**B**) were analyzed by Western blotting. (**C**) The level of TEL mRNA was increased after paeonol treatment by real-time PCR. (**D**,**E**) Paeonol decreased the levels of the SASP markers (IL-6 and TNF-α) in the cell supernatant using ELISA assay. (**F**) The cell proliferation capacity of VSMCs was determined by the EdU staining assay. The results are expressed as mean ± SD, ** *p* < 0.01 versus control; ^#^ *p* < 0.05, ^##^ *p* < 0.01 versus t-BHP.

SIRT1 has been shown to bind to telomeres and contribute to telomere integrity [23], while telomere damage promotes senescence of VSMCs after vessel injury and inflammation. Therefore, we further evaluated the impact of paeonol on SIRT1-mediated telomere damage and inflammation of t-BHP-stimulated VSMCs senescence. As shown in Figure 5A, SIRT1 expression increases significantly after paeonol treatment compared to VSMCs stimulated with t-BHP. Furthermore, paeonol robustly increases the expression of the TRF2 protein and TEL mRNA in t-BHP-stimulated VSMCs (Figure 5B,C), implying that paeonol reduces the damage of the telomere in senescent VSMCs. Production of pro-inflammatory SASP, such as IL-6 or TNF-α, by senescent VSMCs leads to a severe inflammatory reaction to surrounding cells and aorta tissues. We have previously found that paeonol could down-regulate the level of IL-6 and TNF-α in the aorta and serum in vivo. Similarly, our in vitro evaluation provides evidence that paeonol also inhibits the secretion of IL-6 and TNF-α by senescent VSMCs in vitro and alleviates the inflammatory response (Figure 5D,E). Senescence of VSMCs and subsequent more severe inflammatory reactions may lead to decreased proliferation and even death of VSMCs [3]. According to the EdU staining assay, more actively proliferative cells are observed after paeonol treatment (Figure 5F). Therefore, it can be concluded that paeonol effectively improves proliferative capacity and inhibits telomere damage and inflammation of senescent VSMCs.

### 2.6. SIRT1 Is a Potential Cellular Target for Paeonol to Delay the Senescence of VSMCs

Given the inhibitory effects of paeonol on atherosclerosis progression and VSMC senescence, we then tried to explore the potential targets of paeonol. We confirmed the up-regulation of SIRT1 expression by paeonol in vivo and in vitro, and we then explored whether SIRT1 is a key target for paeonol to inhibit VSMC senescence.

**Figure 6:**
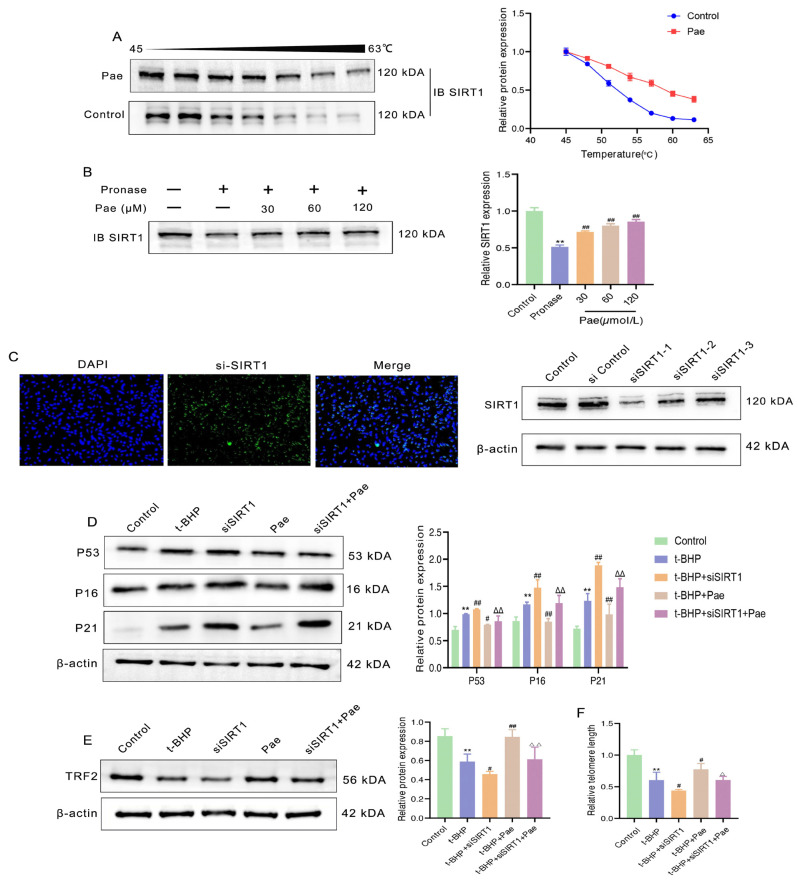
SIRT1 is a potential cellular target of paeonol in VSMCs. (**A**) Paeonol promoted the resistance of SIRT1 to different temperature gradients, which was detected by CETSA in VSMCs. (**B**) Paeonol enhanced the resistance of SIRT1 to proteases, which was investigated by DARTS. (**C**) SIRT1 was successfully silenced in VSMCs by immunofluorescence staining and Western blot. Protein expression levels of senescence-associated proteins (P53, P16, P21) (**D**) and TRF2 (**E**) were analyzed by Western blotting in the control, t-BHP, siSIRT1, paeonol, and siSIRT1 + paeonol groups. (**F**) The level of TEL mRNA was assessed by real-time PCR. The results are expressed as mean ± SD, ** *p* < 0.01 versus control; ^#^ *p* < 0.05, ^##^ *p* < 0.01 versus t-BHP; ^Δ^ *p* < 0.05, ^ΔΔ^ *p* < 0.01 versus paeonol.

After importing target proteins (SIRT1) and compound molecules (paeonol) into AutoDock Vina, we found that paeonol bound SIRT1 with a favorable binding affinity of −6.5 kcal/mol, suggesting that paeonol may theoretically bind to SIRT1 (unpublished data). The CETSA assay was then used to examine whether paeonol binds to SIRT1 in VSMCs. Treatment with paeonol improved the thermal stability of SIRT1, suggesting that paeonol interacts with SIRT1 in VSMCs (Figure 6A). Furthermore, the DARTS experiment revealed that paeonol had the potential effect to stabilize SIRT1, resulting in increased susceptibility to proteolysis (Figure 6B). Our observations supported the evidence that paeonol has an outstanding binding potential with SIRT1 in VSMCs.

To explore whether paeonol can delay VSMC senescence by targeting SIRT1, we constructed VSMCs with low SIRT1 expression using the gene silencing technique. First, we verified whether SIRT1 was successfully silenced in VSMCs by immunofluorescence staining and Western blotting assays. As shown in Figure 6C, siSIRT1 was successfully accumulated in VSMCs, and the protein expression level of SIRT1 was significantly reduced. We selected siSIRT1-1 as the transfection sequence for subsequent experiments. Furthermore, paeonol-mediated inhibition on cell senescence and telomere damage was substantially impaired after SIRT1 depletion (Figure 6D–F). In summary, the results demonstrated that paeonol attenuated the senescence of VSMCs by binding and increasing SIRT1 expression.

### 2.7. Paeonol Attenuates t-BHP-Stimulated VSMCs Senescence by Activating the SIRT1/P53/TRF2 Pathway

**Figure 7:**
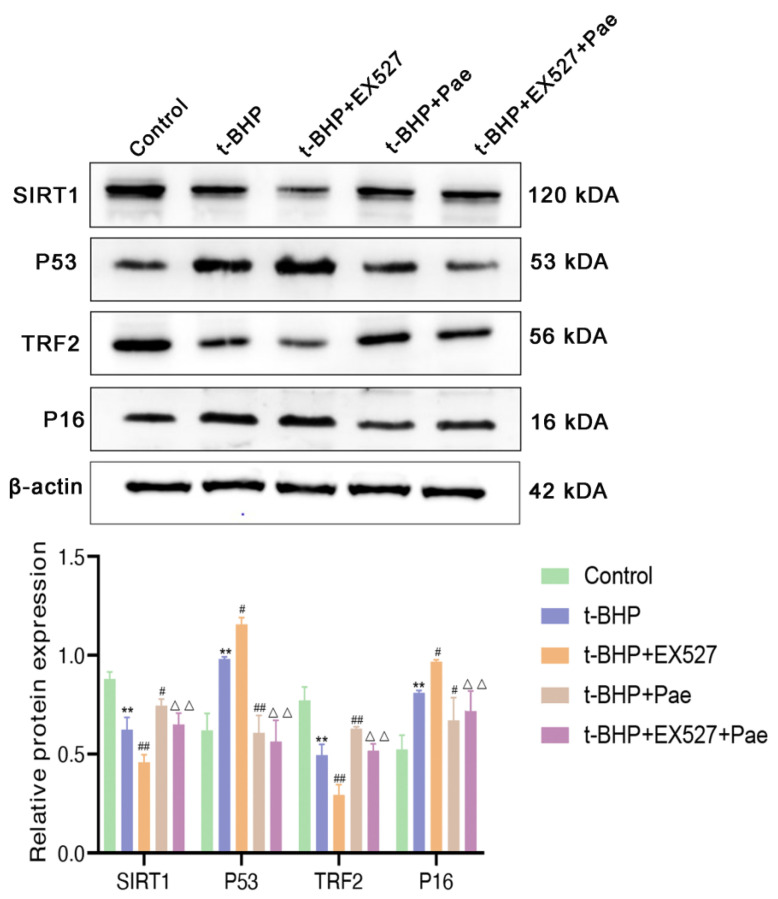
Paeonol inhibits VSMC senescence by activating the SIRT1/P53/TRF2 signaling pathway. The results are expressed as mean ± SD, ** *p* < 0.01 versus control; ^#^ *p* < 0.05, ^##^ *p* < 0.01 versus t-BHP; ^ΔΔ^ *p* < 0.01 versus t-BHP + paeonol.

To elucidate the potential pharmacological mechanism of paeonol in VSMC senescence, we further detected the expression levels of proteins related to the SIRT1 downstream pathway in VSMCs. As shown in Figure 7, t-BHP induced a reduction in SIRT1 and TRF2 and activation of P53 and P16 compared to the control group. However, paeonol treatment effectively inhibited these changes in senescence-associated proteins in VSMCs stimulated with t-BHP. To further confirm whether the inhibitory effects of paeonol on VSMCs senescence were caused by the SIRT1 pathway, the SIRT1 inhibitor EX527 was used. Our results showed that EX527 reversed the protective effects of paeonol on VSMC senescence and telomere damage (Figure 7), suggesting that the SIRT1/P53/TRF2 signaling pathway could be the key signaling pathway mediated by paeonol to alleviate VSMC senescence.

## 3. Discussion

Animal and human studies have suggested that paeonol, the main active compound extracted from the Chinese traditional medicine *Paeonia suffruticosa* Andr., could be used as an anti-inflammatory agent [14]. Atherosclerosis is the common pathological basis for many cardiovascular diseases, and inflammation is a critical pathogenesis and driving factor for atherosclerosis [17]. Our group has long been committed to exploring the anti-inflammatory effects of paeonol to delay the process of atherosclerosis, and confirmed that paeonol could decrease the area of aortic intimal plaque, reduce vascular inflammation [24]. As expected, paeonol significantly inhibited aortic plaque formation in ApoE^−/−^ mice after a 4-week intervention. How paeonol modulates the major pathways that inhibit atherosclerosis progression remains largely unclear.

In this study, we found elevated levels of senescence-associated protein expression in the aorta of ApoE^−/−^ mice, suggesting that the blood vessels of atherosclerotic mice are at least partially senescent. Numerous studies have confirmed the association between vascular senescence and atherosclerosis [25,26]. Our results demonstrated that paeonol significantly inhibited vascular senescence in ApoE^−/−^ mice. SASP produced by senescent cells further aggravates local vascular inflammation and accelerates the progression of atherosclerosis [4]. We observed that paeonol treatment of ApoE^−/−^ mice attenuated vascular senescence represented by reduced SASP markers TNF-α and IL-6, together with a reduction in cellular telomere damage in the aorta, consistent with the pharmacological role of paeonol in reducing vascular inflammation and its anti-atherosclerotic activity. Thus, paeonol played a protective role in atherosclerosis-associated vascular senescence in ApoE^−/−^ mice. It is worth noting that the present study only observed that paeonol has a certain delaying effect on the process of atherosclerosis and the vascular senescence, and we should continue to explore how to increase the therapeutic effect of paeonol to make it have clinical therapeutic potential in future research.

VSMCs play a pivotal role in the progression of atherosclerosis, and aging of some VSMCs in atherosclerotic plaques cause further inflammation of surrounding cells by secreting SASP [19]. Although paeonol has been reported to inhibit vascular endothelial cell senescence [18], whether paeonol can regulate VSMC senescence remains to be clarified. In this study, paeonol treatment alleviated VSMC senescence and inflammation in both ApoE^−/−^ mice and t-BHP-stimulated VSMCs. Importantly, our results revealed that paeonol significantly inhibited the damage to the telomere of VSMCs. Two lines of evidence support this notion. First, paeonol treatment significantly decreased the expression of senescence-related proteins from VSMCs in vivo and in vitro. Second, VSMC-specific overexpression of TRF2 in ApoE^−/−^ mice prevented senescence, reduced telomere damage, and inhibited necrotic core formation. Higher expression of TEL mRNA and TRF2 protein were found after paeonol treatment. However, this study still had some limitations. Cell senescence is related to functions such as cell proliferation, cell cycle, and the inflammatory response. Furthermore, telomere damage has been confirmed in senescent cells. Further studies are required to explore the association between cell senescence, telomere damage, and paeonol activity.

SIRT1 deficiency has been reported to induce cell senescence and accelerate the progression of cardiovascular disease, particularly atherosclerosis [27,28]. Although our findings confirmed that paeonol could inhibit the senescence of VSMCs and delay atherosclerosis processes, the direct target of paeonol involved in the inhibition of VSMC senescence is not fully understood. With advances in drug target-finding techniques [29,30,31], we further explored whether paeonol might work by directly targeting SIRT1. In this study, paeonol, a potential anti-inflammatory agent for the treatment of atherosclerosis, potently enhanced the expression of the SIRT1 protein in vivo and in vitro. Paeonol acted by binding to SIRT1 and increased the expression of the SIRT1 protein in VSMCs, thus promoting the proliferation of senescent cells and reducing the inflammatory reaction. Interestingly, the effects of paeonol on the up-regulation of SIRT1 were reversed in siSIRT1-induced VSMCs, with the concomitant promotion of cell proliferation and suppression of the inflammatory response, suggesting a link between paeonol and SIRT1 signaling.

The signaling mechanism of paeonol that inhibits VSMC senescence is not fully understood. In the present study, the role of the SIRT1 signaling pathway in the inhibition of VSMC senescence was investigated. Inhibition of P53 is achieved by activation of SIRT1, which in turn regulates the expression of downstream TRF2 to affect cell function [32,33]. Here, we found that the protein expression of P53 was significantly down-regulated by paeonol. Deletion of TRF2 is a key mechanism and inducer of telomere shortening and damage. Furthermore, paeonol significantly increased the expression of the TRF2 protein. We speculate that the SIRT1/P53/TRF2 signaling pathway is an important mechanism through which paeonol inhibits the senescence of VSMCs. Furthermore, we found that EX527, a specific inhibitor of SIRT1, strongly reversed the regulatory effects of paeonol on the SIRT1/P53/TRF2 signaling pathway and cell senescence in VSMCs.

## 4. Materials and Methods

### 4.1. Reagents and Antibodies

Paeonol (Cat. No. T1909226; 99% purity) was obtained from Baicao Plants Biotech Co., Ltd. (Bozhou, China). t-BHP (Cat. No. 416665) was purchased from Sigma Aldrich (St. Louis, MO, USA) and EX-527 (Cat. No. HY-15452) was purchased from Med Chem Express (Monmouth Junction, NJ, USA). Dulbecco’s modified eagle medium (DMEM, Cat. No. CA0002) was obtained from SparkJade Science Co., Ltd. (Yantai, China), phosphate buffered saline (PBS, Cat. No. BL601A) and penicillin/streptomycin (Cat. No. BL505A) was obtained from Bioshar (Beijing, China), and fetal bovine serum (FBS, Cat. No. 086-150) was obtained from Wisent Inc. (Nanjing, China). The anti-SIRT1 antibody (Cat. No. R25721) and the goat anti-rabbit IgG secondary antibody (Cat. No. 511203) were obtained from Chengdu Zen Bioscience Co., Ltd. (Chengdu, China). The P53 antibody (Cat. No. TA0879F) was obtained from Abmart (Shanghai, China). The TERT antibody (Cat. No. Df7129) was obtained from Affinity Bioscience (Changzhou, China). The polyclonal antibody P21 (YT3497), the p16 polyclonal antibody p16 (YT3493), and the polyclonal antibody TERF2 (YN0016) were obtained from Immunoway (Beijing, China).

### 4.2. Animal Experiments

Male ApoE^−/−^ mice weighing 17 and 27 g were obtained from Qizhen Laboratory Animal Co., Ltd. (Hangzhou, China) and housed in a room kept at 22 ± 2 °C and relative humidity 50% ± 5%. After a week of random adaptation with food and water, mice were fed a HFD (composed of 40 kcal% fat-derived and 0.15% cholesterol chow) for 12 weeks until atherosclerotic lesions were formed in the arteries, and then the mice were randomly divided into five groups: (1) normal diet (Control), (2) HFD (Model), (3) HFD + paeonol (200 mg/kg body weight), (4) HFD + paeonol (400 mg/kg body weight), (5) HFD+ simvastatin (25 mg/kg body weight, SIM) (*n* = 8/group). Mice received 0.5% CMC-Na solution containing 200, and 400 mg/kg paeonol by gavage for 4 weeks, while C57BL/6 mice in the control group received 0.5% CMC-Na solution without paeonol by gavage.

### 4.3. Cell Culture and Treatment

VSMCs were isolated from C57BL/6J mouse arteries as described in our previous study [16]. VSMCs were then maintained at 37 °C in a 5% CO_2_ atmosphere and cultured in complete DMEM (supplemented with 10% heat inactivated FBS and 1% penicillin/streptomycin). The cells were passed when 85% confluence was achieved. To investigate the effects of paeonol on VSMC senescence, VSMCs were treated with paeonol (30, 60, 120 μM) for 24 h with or without 80 μM t-BHP.

### 4.4. Analysis of Atherosclerotic Lesions

According to previous research by our group, we used Oil-Red O (ORO) staining and hematoxylin and eosin (H&E) staining to detect the aortic plaques. We carefully excised the entire artery, including the aortic arch, chest, and abdomen along the midline, which was followed by ORO staining. In H&E staining, aortic tissue was first processed into paraffin-embedded blocks, then cut into 5 μm slices for subsequent staining. Finally, ImageJ was used to analyze the area of the lesion.

### 4.5. Micro-Ultrasound

The micro-ultrasound parameters of the left common carotid artery and abdominal aorta were measured using the Silicon Wave 60 system (Kolo, Beijing, China) after 12 weeks of HFD stimulation in ApoE^−/−^ mice. In brief, mice were anesthetized by inhalation of 2% isoflurane. Neck fur was defoliated, and ultrasound gel was applied liberally. A scan head of 30 MHz was applied to measure luminal diameters and intimal–medial thickness in each group.

### 4.6. Cell Counting Kit-8 (CCK-8) Assay

The cytotoxic effects of paeonol were determined by CCK-8 assay. Briefly, cells were seeded in a 96-well plate at the optimal cell density. After the cells were incubated overnight, they were pretreated with paeonol (30, 60, 120 μM) for 12, 24, and 36 h. The cells were then incubated with 10 μL of CCK-8 solution for 2 h in the dark at 37 °C, in a 5% CO_2_ incubator. The absorbance at 450 nm was measured using a microplate reader.

### 4.7. ELISA Assay

In the animal experiment, the aorta was removed and was fully ground with a grinder at 5000 r/min, centrifuged for 15 min, and then the supernatant was collected. Cell experiments were divided into the following groups: control group (in DMEM), t-BHP group, and different concentrations of paeonol (30, 60,120 μM) with t-BHP for 24 h. Supernatants from the above groups were collected and TNF-α and IL-6 were detected by ELISA kits according to the manufacturer’s instructions.

### 4.8. Immunofluorescence Staining

First, the sample was fixed with 4% paraformaldehyde, embedded in paraffin, and cut into thin slices of approximately 20 microns. Next, 0.1% Triton X-100 was infiltrated in PBS and sealed with 0.5% BSA. Then, the samples were exposed to primary antibodies SA-β-Gal (1:500) and α-SMA (1:500) and incubated overnight at 4 °C. Then, secondary antibodies Alexa Fluor 488 rabbit anti-rat IgG (H + L) (Zen Bioscience, Cat. EF0010, 1:100) and Alexa Fluor 594 goat anti-mouse IgG (H + L) (Spark Jade, Cat. 550067, 1:100) were incubated at room temperature for 2 h, followed by repeated washing with PBS. After staining the nucleus with DAPI for 15 min, images were recorded using a fluorescence microscope (DM24000B, Leica, Wetzlar, Germany).

### 4.9. Quantitative Real-Time PCR Assay

According to the TIANamp Genomic DNA Kit (Beijing, China), the extracted RNA from the aorta and cell DNA were adjusted to 20 mg/L. The primer sequences for the telomere (TEL) and reference (AT1) genes were as follows: TEL forward primer 5′-GGTTTTTGAGGGTGAGGGTGAGGGTGAGGGTGAGGGTGAGGGTGAGGGTGAGGGT-3′, TEL reverse primer 5′-TCCCGACTATCCCTATCCCTATCCCTATCCCTATCCCTATCCCTATCCCTATCCCTA-3′, AT1 forward primer 5′-ACGTGTTCTCAGCATCGACCGCTACC-3′, and AT1 reverse primer 5′-AGAATGATAAGGAA-AGGGAACAAGAAGCCC-3′.

### 4.10. EdU Staining

Cell proliferation ability was analyzed by EdU staining; multiple fields of view were randomly captured using a fluorescence microscope (DM24000B, Leica). The captured images allowed the distinction of EdU-positive nuclei (green) and total nuclei (stained with DAPI, blue). The EdU-positive cell rate was determined as the ratio of EdU-positive nuclei (green) to the total number of nuclei (blue fluorescent) in the captured fields of view. This analysis provided a quantitative assessment of cell viability and proliferation.

### 4.11. Cellular Thermal Shift Assay

To evaluate the thermal stability of the SIRT1 protein, we conducted the cellular thermal shift assay (CETSA). For Western blotting, total protein extraction was performed. Subsequently, the supernatant was evenly divided into two centrifuge tubes, and equal volumes of DMSO and paeonol were added separately. The samples were then incubated at room temperature for 1 h. After incubation, a temperature gradient was applied, with stepwise heating at 45, 48, 51, 54, 57, 60, and 63 °C for 3 min, followed by cooling to room temperature, and the supernatants were detected by Western blotting.

### 4.12. Drug Affinity Responsive Target Stability

The VSMCs were lysed in a 500 μL RIPA lysis buffer (50 μL PMSF) to perform the drug affinity responsive target stability assay (DARTS). The lysate was divided into five identical EP tubes and incubated with paeonol (0, 30, 60 and 120 μM) for 1 h at room temperature. The sample was hydrolyzed with collagenase E (1 μg/mL) in a reaction buffer for 10 min. A protein loading buffer was added to stop the reaction, and the specific anti-SIRT1 antibody was used for Western blotting detection.

### 4.13. SiRNA Silencing SIRT1

VSMCs were cultured to a confluence of 85% prior to transfection with small interference RNAs (siRNAs) targeting SIRT1 according to the manufacturer’s instructions (Suzhou, China). The SIRT1 siRNA sequences used were as follows: 5′-GUGGCAGAUUGUUAUUAAUTT-3′, 5′-CCCAUGAAGUGCCUCAAAUTT-3′, and 5′-GUGGUGAAUAUGCCAAACUTT-3′, and supernatants were used for Western blotting.

### 4.14. Western Blotting

VSMCs and aorta tissue were lysed in a RIPA lysis buffer. The BCA protein assay kit was used to measure protein concentration. Equal amounts of protein were then fractionated on SDS-PAGE and then transferred to PVDF membranes. Primary antibodies against SIRT1 (1:1500), P53 (1:2000), P21 (1:2000), P16 (1:2000), TRF2 (1:1000), and β-actin (1:10,000) were incubated with the membranes at 4 °C overnight. Subsequently, the membranes were exposed to HRP-labeled secondary antibody (1:10,000, Beyotime, Shanghai, China), and Ultra-Sensitive Multifunctional Imager (GE, Salt Lake, UT, USA) was utilized for signal detection.

### 4.15. Statistical Analysis

All data are presented as mean ± SD, and SPSS v.23.0 was used for statistical analysis. Student’s *t* test was used for statistical comparisons between the two groups, while one-way analysis of variance (ANOVA) was used for multiple groups. A value of *p* < 0.05 or *p* < 0.01 was considered statistically significant.

## 5. Conclusions

In our study, using in vitro and in vivo models of atherosclerosis-induced VSMC senescence, we provide evidence that paeonol treatment produces a significant inhibitory effect on VSMCs senescence and telomere damage through the SIRT1/P53/TRF2 signaling pathway. The present study provides new information on the potential of paeonol treatment in the prevention of VSMC senescence and atherosclerosis and suggests that paeonol may be a promising candidate as a SIRT1 agonist for the treatment of atherosclerosis.

## Data Availability

The data presented in this study are available on request from the corresponding author. The data used in this study, including molecular docking, are not publicly available due to confidentiality.

## Abbreviations

ApoE^−/−^, Apolipoprotein E knockout mice; CETSA, Cellular thermal shift assay; CCK8, Cell counting kit-8; DAPI, 4′,6-diamidino-2-phenylindole; DARTS, Drug affinity responsive target stability assay; H&E, Hematoxylin and eosin; HFD, High-fat diet; ORO, Oil-Red O; siRNAs, Small interference RNAs; SIRT1, Sirtuin 1; TRF2, t-BHP, tert-butyl hydroperoxide; Telomeric repeat binding factor-2; VSMCs, Vascular smooth muscle cells.
